# Simultaneous feature selection and parameter optimisation using an artificial ant colony: case study of melting point prediction

**DOI:** 10.1186/1752-153X-2-21

**Published:** 2008-10-29

**Authors:** Noel M O'Boyle, David S Palmer, Florian Nigsch, John BO Mitchell

**Affiliations:** 1 Unilever Centre for Molecular Science Informatics, Dept. of Chemistry, University of Cambridge, Lensfield Rd, Cambridge, CB2 1EW, UK; 2 Cambridge Crystallographic Data Centre, 12 Union Rd, Cambridge, CB2 1EZ, UK; 3 Department of Chemistry, Aarhus University, 8000 Aarhus C, Denmark

## Abstract

**Background:**

We present a novel feature selection algorithm, Winnowing Artificial Ant Colony (WAAC), that performs simultaneous feature selection and model parameter optimisation for the development of predictive quantitative structure-property relationship (QSPR) models. The WAAC algorithm is an extension of the modified ant colony algorithm of Shen et al. (*J Chem Inf Model *2005, **45**: 1024–1029). We test the ability of the algorithm to develop a predictive partial least squares model for the Karthikeyan dataset (*J Chem Inf Model *2005, **45**: 581–590) of melting point values. We also test its ability to perform feature selection on a support vector machine model for the same dataset.

**Results:**

Starting from an initial set of 203 descriptors, the WAAC algorithm selected a PLS model with 68 descriptors which has an RMSE on an external test set of 46.6°C and R^2 ^of 0.51. The number of components chosen for the model was 49, which was close to optimal for this feature selection. The selected SVM model has 28 descriptors (cost of 5, ε of 0.21) and an RMSE of 45.1°C and R^2 ^of 0.54. This model outperforms a *k*NN model (RMSE of 48.3°C, R^2 ^of 0.47) for the same data and has similar performance to a Random Forest model (RMSE of 44.5°C, R^2 ^of 0.55). However it is much less prone to bias at the extremes of the range of melting points as shown by the slope of the line through the residuals: -0.43 for WAAC/SVM, -0.53 for Random Forest.

**Conclusion:**

With a careful choice of objective function, the WAAC algorithm can be used to optimise machine learning and regression models that suffer from overfitting. Where model parameters also need to be tuned, as is the case with support vector machine and partial least squares models, it can optimise these simultaneously. The moving probabilities used by the algorithm are easily interpreted in terms of the best and current models of the ants, and the winnowing procedure promotes the removal of irrelevant descriptors.

## Background

Quantitative Structure-Activity and Structure-Property Relationship (QSAR and QSPR) models are based upon the idea, first proposed by Hansch [1], that a molecular property can be related to physicochemical descriptors of the molecule. A QSAR model for prediction must be able to generalise well to give accurate predictions on unseen test data. Although it is true in general that the more descriptors used to build a model, the better the model predicts the training set data, such a model typically has very poor predictive ability when presented with unseen test data, a phenomenon known as overfitting [2]. Feature selection refers to the problem of selecting a subset of the descriptors which can be used to build a model with optimal predictive ability [3]. In addition to better prediction, the identification of relevant descriptors can give insight into the factors affecting the property of interest.

The number of subsets of a set of *n *descriptors is 2^n^-1. Unless *n *is small (<20) it is not feasible to test every possible subset, and the number of descriptors calculated by cheminformatics software is usually much larger (CDK [4], MOE [5] and Sybyl [6] can respectively calculate a total of 95, 146 and 248 1D and 2D descriptors). Feature selection methods can be divided into two main classes: the filter approach and the wrapper approach [3,7,8]. The filter approach does not take into account the particular model being used for prediction, but rather attempts to determine *a priori *which descriptors are likely to contain useful information. Examples of this approach include ranking descriptors by their correlation with the target value or by estimates of the mutual information (based on information theory) between each descriptor and the response. Another commonly used filter in QSAR is the removal of highly correlated (or anti-correlated) descriptors [9]. Liu [10] presents a comparison of five different filters in the context of prediction of binding affinities to thrombin. The filter approach has the advantages of speed and simplicity, but the disadvantage that it does not explicitly consider the performance of the model containing different features. Correlation criteria can only detect linear dependencies between descriptor values and the response, but the best performing QSAR models are often non-linear (support vector machines (SVM), neural networks (NN) and random forests (RF), for example). In addition, Guyon and Elisseeff show that very high correlation (or anti-correlation) does not necessarily imply an absence of feature complementarity, and also that two variables that are useless by themselves can be useful together [3].

The wrapper approach conducts a search for a good feature selection using the induction algorithm as a black box to evaluate subsets and calculate the value of an objective function. The objective function should provide an estimate of how well the model will generalise to unseen data drawn from the same distribution. The purpose of the search is to find the feature selection that optimises this value. The most well-known deterministic wrapper is sequential forward selection [11] (SFS) which involves successive additions of the feature that most improves the objective function to the subset of descriptors already chosen. A related algorithm, sequential backwards elimination [12] (SBE), successively eliminates descriptors starting from the complete set of descriptors. Both of these algorithms suffer from the problem of 'nesting'. In the case of SFS, nesting refers to the fact that once a particular feature is added it cannot be removed at a later stage, even if this would increase the value of the objective function. More sophisticated methods, such as the sequential forward floating selection (SFFS) algorithm of Pudil et al. [13], include a backtracking phase after each addition where variables are successively eliminated if this improves the objective function. Wrapper methods specific to certain models have also been developed. For example, the Recursive Feature Elimination algorithm of Guyon et al. [14] and the Incremental Regularised Risk Minimisation of Fröhlich et al. [15] are specific to models built using support vector machines.

Stochastic wrappers attempt to deal with the size of the search space by incorporating some degree of randomness into the search strategy. The most well known of these algorithms is the genetic algorithm [16] (GA), whose search procedure mimics the biological process of evolution. A number of models are created randomly in the first generation, the best of which (as measured by the objective function) are selected and interbred in some way to create the next generation. A mutation operator is applied to the new models so that random sampling of the local space occurs. Over the course of many generations, the objective function is optimised. Genetic algorithms were first used for feature selection in QSAR by Rogers and Hopfinger [17] and are now used widely [9,18,19]. Other stochastic methods which have been used for feature selection in QSAR are particle swarm optimisation [20,21] and simulated annealing [22].

An additional difficulty in the development of QSAR models is the fact that some regression methods have parameters that need to be optimised to obtain the best performance for a particular problem. The Support Vector Machine (SVM) is an example of such a method. A SVM is a kernel-based machine learning method used for both classification and regression [23-25] which has shown very good performance in QSAR studies [9]. In ε-SVM regression, the algorithm finds a hyperplane in a transformed space of the inputs that has at most ε deviation from the output y values. Deviations greater than ε are penalised by multiplying by a cost value C. The transformation of the inputs is carried out by means of kernel functions, which allows nonlinear relationships between the inputs and the outputs to be handled by this essentially linear method. For a particular problem and kernel, the values of C and ε must be tuned.

Here we describe WAAC, Winnowing Artificial Ant Colony, a stochastic wrapper for feature selection and parameter optimisation that combines simultaneous optimisation of the selected descriptors and the model parameters to create a model with good predictive accuracy. This method does not require any pre-processing of the data apart from removal of zero-variance and duplicate descriptors. The only requirement is that allowed values of parameters of the models must be specified. As a result, this method is suitable for use as an automatic generator of predictive models.

The WAAC algorithm is a novel stochastic wrapper derived from the modified Ant Colony Optimisation (ACO) algorithm of Shen *et al. *[26]. Ant colony algorithms take their inspiration from the foraging of ants whose cooperative behaviour enables the shortest path between nest and food to be found [27]. Ants deposit a substance called pheromone as they walk, thus forming a pheromone trail. At a branching point, an ant is more likely to choose the trail with the greater amount of pheromone. Over time as pheromones evaporate, only those trails that have been reinforced by the passage of many ants will retain appreciable amounts of pheromone, with the shortest trail having the greatest amount of pheromone. In the end, all of the ants will travel by the shortest trail. Artificial ant colony systems may be used to solve combinatorial optimisation problems by making use of the ideas of cooperation between autonomous agents through global knowledge and positive feedback that are observed in real ant colonies [28].

The first use of artificial ant systems for variable selection in QSAR was the ANTSELECT algorithm of Izrailev and Agrafiotis [29]. The ANTSELECT algorithm involves the movement of a single ant through feature space. Initially equal weights are assigned to each descriptor. The probability of the ant choosing a particular descriptor in the next iteration is the weight for that descriptor divided by the sum of all weights. After the fitness of the model is assessed, all of the weights are reduced by multiplying by (1-ρ), where ρ is the evaporation coefficient. The weights of those descriptors selected in the current iteration are then increased by a constant multiple of the fitness score. Gunturi *et al. *[30] used a modification of the ANTSELECT algorithm in a recent study of human serum albumin binding affinity in which the number of features selected was fixed *a priori *and, in addition, could not include descriptors that had a correlation coefficient greater than 0.75.

Since the ANTSELECT algorithm uses only a single ant, it cannot make use of one of the most important features of ant colony algorithms, collective intelligence. Instead, premature convergence will occur due to positive reinforcement of models that have performed well earlier in the local search. In addition, the search space will be poorly covered. Although the authors recommend that the algorithm should be repeated several times to minimise the likelihood of convergence to a poor local minimum, the use of an ant colony is a much more robust solution.

Shen *et al*. [26] presented an ACO algorithm that differed from ANTSELECT in several ways. Their algorithm, which they called a modified ACO, is similar to our WAAC algorithm in that it involves a colony of ants, each of which remembers its best model and score, as well as its current model and score. In Shen *et al.*'s algorithm, for every descriptor there are both positive and negative weights. The probability that an ant will choose a particular descriptor is given by the positive weight for that descriptor divided by the sum of the positive and negative weights. After every iteration, the weights are reduced by multiplying by (1-ρ) as for ANTSELECT. The positive weight for a particular descriptor is increased by the sum of the fitness scores of all ants in the current iteration that have selected it, as well as the fitness scores of the best models of all ants that have selected it in that model. Similarly, the negative weight for a particular descriptor is decreased by an amount based on the fitness scores of models that have not selected it.

In the following section, we describe the WAAC algorithm in detail, as well as the dataset and model used to test the algorithm. In the Results and Discussion sections, we describe the performance of the WAAC algorithm, compare it to other models on the same dataset, and discuss some practical considerations in usage.

## Methods

### WAAC algorithm

The WAAC algorithm uses a population of candidate models termed an 'ant colony'. Each ant represents a model; that is, it is associated with a particular feature selection as well as particular values for the model (for example, SVM) parameters. The set of descriptors is stored as a binary fingerprint of length F (the number of descriptors), where a value of 1 for the n^th ^bit indicates that the n^th ^descriptor is selected, and 0 indicates that it is not. For each parameter of the model, a range of discrete values is required. The parameter values used by a particular ant are stored in a list of length P, where P is the number of adjustable parameters of the model. The fitness of each model is measured using an objective function specified by the user.

The initial population of ants is randomly placed in feature and parameter space. The bits of the binary fingerprints representing the feature selections are initialised to either 0 or 1 with equal probability, so that on average each ant corresponds to a model based on approximately 50% of the descriptors. Conversely, each descriptor is initially selected by approximately 50% of the ants. The initial parameter values for each ant are chosen at random from the available values for each parameter.

**Figure 1:**
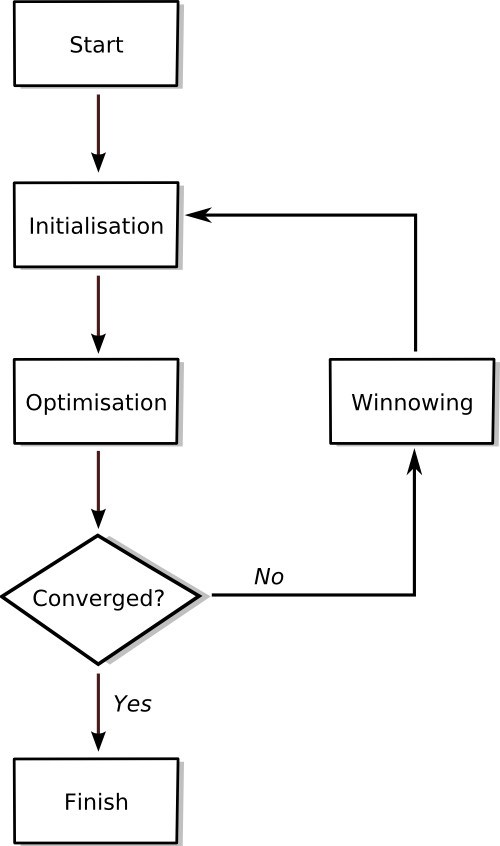
Outline of the WAAC algorithm.

Figure 1 shows a schematic of the WAAC algorithm. After initialisation, the algorithm enters the optimisation phase. For each descriptor, a moving probability is calculated by taking the average of the fraction of ants which have currently selected that descriptor and the fraction that have selected that descriptor in their best model. This moving probability is used to determine the chance that a particular ant will select a particular descriptor in the next iteration. At the start of the optimisation phase, the moving probabilities for all of the descriptors will be approximately equal to 0.5 (since the best model will be the current model and each descriptor is selected by approximately 50% of the ants).

Similarly, for each parameter there is a moving probability associated with every allowed value. These moving probabilities sum to unity (since each ant needs to select exactly one allowed value for each parameter), and are calculated by taking the average of the fraction of ants which have currently selected a particular allowed value and the fraction of ants that have selected that value in their best model. At the start of the optimisation phase, each allowed value of a parameter will be selected by approximately N/P ants where N is the number of ants, and P the number of allowed values.

At the start of the optimisation phase, the ants move more or less randomly, as the moving probabilities are essentially equal for all features and parameter values. However, over the course of the optimisation phase as particular descriptors are found to occur frequently in the best models associated with the ants, due to positive feedback these descriptors will be more likely to be chosen in subsequent iterations. This global optimisation procedure is combined with local optimisation due to the influence of the current positions of the ants on the moving probabilities. Note that the ants do not move about relative to their position in a previous iteration; rather, their subsequent location in feature space is determined by the best and current feature selections of all of the ants. Note that nesting is not a problem, as in each step of the optimisation the ants are free to explore descriptor combinations which did not exist in the previous step.

After multiple iterations of the optimisation algorithm, a winnowing procedure is applied. This reduces the search space by retaining only those descriptors that have been chosen by at least 20% of the ants in their best models, and removing the rest. Parameter values are reinitialised randomly. Some descriptors may be retained that do not improve the models, but the subsequent reinitialisation of the ants on the smaller search space will allow the subsequent optimisation phase to identify better models which exclude that descriptor. Note that no information is carried from one optimisation procedure to the next. In particular, memory of previous best models does not guide future searching. This means that the randomly initialised models in the new optimisation phase are always poorer than the best models of the previous phase, but the reduction in the size of the feature space means that the performance of the model quickly recovers and matches or improves on earlier performance.

As shown in Figure 1, the optimisation phase and winnowing procedure are repeated until convergence is achieved or a specific number of iterations have occurred. The best model found at any point in the entire optimisation procedure should be chosen as the final best model. An implementation of WAAC in R [31] is available from the authors on request.

### Dataset

We use the Karthikeyan dataset [32] of melting point values as described in Nigsch et al. [33]. This is a dataset of melting points of 4119 diverse organic molecules which cover a range of melting points from 14 to 392.5°C, with a mean of 167.3°C and a standard deviation of 66.4°C. Each molecule is described by 203 2D and 3D descriptors, which is the full range of descriptors available in the software MOE 2004.03 [5].

The dataset was randomly divided 2:1 into training data and an external test set (1373 molecules, see additional file 1: externaltest.csv for the original data). The training data was further randomly divided 2:1 into a training set used for model building (1831 molecules, see additional file 2: internaltraining.csv) and an internal test set (915 molecules, see additional file 3: internaltest.csv).

### Objective function

The goal of the WAAC algorithm is to find the feature subset and parameter values that will give the best predictive accuracy for a model based on given training data. During the course of the optimisation, the algorithm needs to be guided by an objective function that will give an estimate of the predictive accuracy of a particular model.

Here we examine the performance of the WAAC algorithm on the Karthikeyan dataset using as our objective function the root mean squared error of the predictions on the internal test set, RMSE(int). Each model is built on the training set using whatever features and parameter values have been selected, and then used to predict the melting point values for the internal test set.

### Statistical testing

To assess the quality of a model, we report three statistics: the squared correlation coefficient, R^2^, the Root-Mean-Square-Error, RMSE, and the bias. These are defined in Equations 1 to 3. A parenthesis nomenclature is used to indicate whether the statistic refers to a model tested on the entire training data (tr) (this includes the internal test set), the internal test set only (int), or the external test set (ext).

(1) $$RMSE=\sqrt{\frac{1}{n}\sum_{i=1}^{n}{\left(y_{i}^{obs}-y_{i}^{pred}\right)}^{2}}$$

(2) $$R^{2}=1-\sum_{i=1}^{n}{\left(y_{i}^{obs}-y_{i}^{pred}\right)}^{2}/\sum_{i=1}^{n}{\left(y_{i}^{obs}-{\bar{y}}^{obs}\right)}^{2}$$

(3) $$bias=\frac{1}{n}\sum_{i=1}^{n}\left(y_{i}^{obs}-y_{i}^{pred}\right)$$

In the prediction of the external test set, an outlier is defined as any point with a residual greater than 4 standard deviations from the mean.

### Models

We used the WAAC algorithm to simultaneously optimise the chosen features and number of components in a Partial Least Squares (PLS) model. The *plsr *method in the *pls *package in R [31] was used to build the PLS model. Scaling was set to true. A range of 20 allowed parameter values for the number of components in the model was initially set to cover from 1 to 191 inclusive in steps of 10. After each winnowing, the step size was reset so that the maximum value for the number of components was less than the number of remaining descriptors. For the WAAC algorithm itself, a colony of 50 ants was used, and the algorithm was run for 800 iterations with winnowing every 100 iterations. For comparison, the algorithm was run for the same length without any winnowing.

In addition, we used the WAAC algorithm to optimise a Support Vector Machine (SVM) model. The *svm *method in the *e1071 *package in R [31] was used to perform ε-regression with a radial basis function. A range of allowed parameter values for the SVM were chosen based on a preliminary run: values for C from 1 to 31 inclusive in steps of 2, and values of ε from 0.01 to 1.61 inclusive in steps of 0.1. Since two parameters needed to be optimised for this model, the length of each optimisation phase in the WAAC algorithm was extended to 150 iterations and the algorithm was run for 1500 iterations in total.

To compare to other feature selection methods, we used the training data to build a Random Forest model [34] using the *randomForest *package in R (using the default settings of *mtry *= *N*/3, *ntree *= 500, *nodesize *= 5). We also compared to the best of thirteen *k *Nearest Neighbours (*k*NN) models trained on the training set, where *k *was 1, 5, 10 or 15. For the models based on multiple neighbours, separate models were created where the predictions were combined using exponential, geometric, arithmetic, or inverse distance weighting (for more details, see Nigsch et al. [33]). The best performing model, as measured by leave-one-out cross validation on the training data, was the 15 NN model with exponential weighting. Hereafter, this model is referred to as the *k*NN model.

### Genetic algorithm

For comparison with the WAAC algorithm, a genetic algorithm for feature selection was implemented in the R statistical programming environment [31]. 50 chromosomes were randomly initialised so that each chromosome on average corresponded to a model based on half of the descriptors. A selection operator chose 10 chromosomes using tournament selection with tournaments of size 3. Once selected, that chromosome was removed from the pool for further selection. A crossover operator was applied to the selected chromosomes, as a single-point crossover between randomly selected (with replacement) chromosomes yielding a pair of children in each case. Each child was subject to a mutation operator which, for a given bit on a chromosome, had a probability of 0.04 of flipping it. The process of crossover and mutation was repeated until 50 offspring were created. The next generation was then formed by the 25 best chromosomes in the original population along with the best 25 of the offspring.

## Results

**Figure 2:**
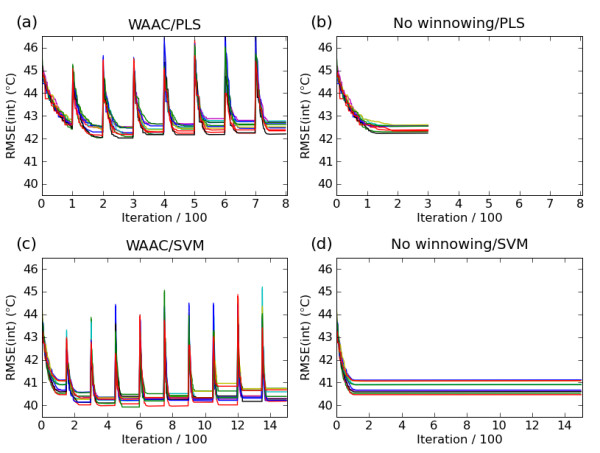
**Value of the objective function for the best model at each iteration of the WAAC algorithm for the PLS model (top) and the SVM model (bottom)**. The figures on the right, (b) and (d), show the effect of having a single optimisation phase without any winnowing. Ten repetitions of the algorithm are shown, with corresponding repetitions starting from the same initial random seed.

The WAAC algorithm was used to search parameter and feature space for a predictive SVM model for the Karthikeyan dataset for both a PLS model and an SVM model. Figures 2(a) and 2(c) show the progress of the algorithm for the PLS and SVM models respectively, as measured by the value of the objective function for the best model found so far in a particular optimisation phase. Each experiment was performed 10 times with different random seeds. For each repetition, the model with the lowest value of the objective function was chosen from among the best models found in each optimisation phase. Of these ten models, the one with the fewest descriptors was chosen as the single final model. This reduces the possibility of finding by chance a model which had an optimal value of the objective function but poor predictive ability.

**Figure 3:**
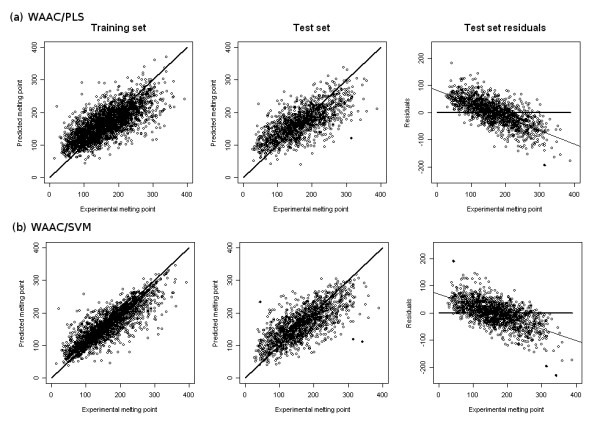
**Performance of models developed with WAAC: (a) a PLS model and (b) an SVM model**. The first two columns contain predictions for the training set and test set, respectively. The line *x *= *y *is shown for comparison. The column on the right shows the residuals from the test set prediction along with a line of best fit (light line); for comparison, the line *x *= 0 is shown (heavy line). Outliers are shown as filled circles in the test set prediction and residuals plots. All values in °C.

**Figure 4:**
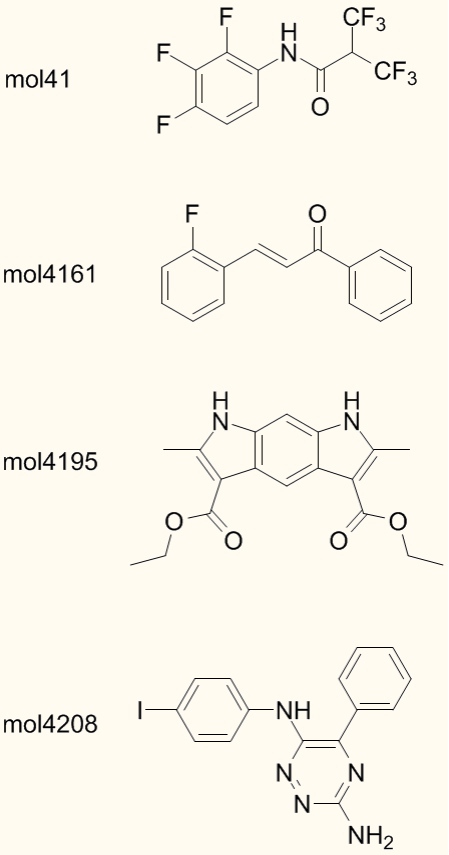
**Structures of outliers for the models discussed in the text**. An outlier is defined as any molecule with a residual greater than four standard deviations from the mean. Molecules 41, 4161 and 4195 are outliers for the WAAC/SVM model; molecules 4161 and 4208 are outliers for both the RF and *k*NN models; molecule 4161 is the single outlier to the WAAC/PLS model.

The selected models for WAAC/PLS and WAAC/SVM are shown in Table 1. Of the 203 original descriptors, only 68 were selected for the PLS model, and 28 for the SVM model. The final models were evaluated by training on the entire training data of 2746 molecules, and predicting the melting point value of the external test set. The results are shown in Figure 3 and summarised in Table 2. The summary statistics for the PLS model are: for the training set, RMSE(tr) = 44.4°C, R^2^(tr) = 0.52, bias = -0.0°C; for the test set, RMSE(ext) = 46.6°C, R^2^(ext) = 0.51, bias = -0.74°C. For comparison, the value of the objective function RMSE(int) was 42.8°C. There was a single outlier, *mol4161 *(Figure 4). The summary statistics for the SVM model are: for the training set, RMSE(tr) = 30.7°C, R^2^(tr) = 0.77, bias = -1.6°C; for the test set, RMSE(ext) = 45.1°C, R^2^(ext) = 0.54, bias = -2.1°C. The value of the objective function RMSE(int) was 40.2°C. Three molecules were identified as outliers to the model: *mol41*, *mol4161 *and *mol4195*. These are drawn as filled circles in Figure 3, and their structures are shown in Figure 4.

**Table 1:** Description of the best models found by the WAAC algorithm

	WAAC/PLS	WAAC/SVM
Number of descriptors	68	28

2D descriptors	petitjean, weinerPath, weinerPol, a_ICM, b_1rotR, chi0_C, chi1, reactive, a_heavy, a_nH, a_nF, a_nO, a_nS, VadjEq, VadjMa, balabanJ, PEOE_RPC+, PEOE_VSA+3, PEOE_VSA+4, PEOE_VSA+5, PEOE_VSA+6, PEOE_VSA-1, PEOE_VSA-4, PEOE_VSA_FPNEG, PEOE_VSA_PPOS, PC+, PC-, Q_PC+, Q_RPC+, Q_VSA_FHYD, Q_VSA_FNEG, Q_VSA_FPNEG, Q_VSA_FPOL, Q_VSA_FPOS, Q_VSA_FPPOS, Q_VSA_PNEG, Q_VSA_PPOS, Kier1, Kier3, KierA1, KierA2, apol, vsa_acc, SlogP_VSA3, SlogP_VSA5, SMR_VSA3, SMR_VSA5, TPSA	radius, weinerPol, b_1rotR, b_rotR, chi1v_c, a_nO, a_nP, balabanJ, PEOE_VSA+2, PEOE_VSA+3, PEOE_VSA-1, PEOE_VSA-5, PEOE_VSA-6, Q_RPC+, SlogP_VSA1, SlogP_VSA4, SlogP_VSA9, SMR_VSA2, SMR_VSA4, SMR_VSA6, TPSA

3D descriptors	AM1_dipole, AM1_Eele, E_sol, E_strain, E_tor, MNDO_HF, MNDO_dipole, MNDO_E, dipole, PM3_HF, ASA-, ASA_H, CASA-, FASA_H, FASA_P, VSA, glob, std_dim1, std_dim3, vol	E_oop, E_strain, E_vdw, PM3_LUMO, FASA_P, FCASA+, rgyr

Parameters	components = 49	Cost = 5, ε = 0.21

**Table 2:** Summary statistics for the models discussed in the text

	WAAC/PLS	WAAC/SVM	SVM	*k*NN	Random Forest
*Training set*					
RMSE (°C)	44.4	30.7	36.2	47.6	17.8 (44.7)*
R^2^	0.52	0.77	0.68	0.44	0.92 (0.51)*
bias (°C)	0.0	-1.6	-2.3	-3.4	0.0
*Test set*					
RMSE (°C)	46.6	45.1	43.9	48.3	44.5
R^2^	0.51	0.54	0.56	0.47	0.55
bias (°C)	-0.7	-2.1	-2.3	-4.1	-0.4
mean (°C)	166.5	165.2	165.0	163.2	167.0
standard deviation (°C)	47.1	51.6	49.3	49.5	41.0

*Line of best fit through test set residuals*					
Slope	-0.49	-0.43	-0.44	-0.49	-0.53

* Out-of-bag estimates for RMSE and R^2 ^are shown in parenthesis.

**Figure 5:**
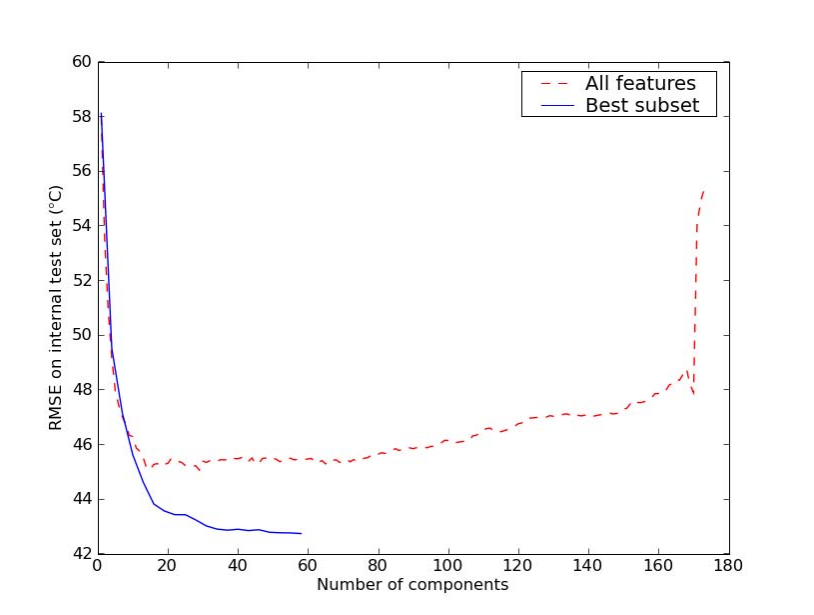
**The effect of the number of components on the predictive ability of a PLS model**. The red dashed line is a model based on all of the features, whereas the model represented by the blue solid line is based only on the subset selected by the WAAC algorithm. The best subset line ends at 59 components, as there are only 59 features in this subset. The line for all features is truncated at 174 components as the RMSE rapidly increases after this point.

For the PLS model the optimised number of components was 49. In order to assess whether the WAAC algorithm sufficiently explored parameter space, we carried out a parameter scan across all allowed values for the parameter with the feature selection found in the best model, and calculated the value of the objective function, RMSE(int). As shown in Figure 5 (solid line), the value of the objective function obtained with 49 components is almost at the minimum, although three larger values for the number of components give slightly better models (42.78°C RMSE(int) versus 42.73°C). For the SVM model, the optimised parameter values associated with the selected model were a cost value of 5, and a value for ε of 0.21. When we carried out a parameter scan across all allowed values of the cost and ε (272 models in total), only one scored higher than the best model, and even then, only marginally: 40.22°C RMSE(int) for cost = 5 and ε = 0.11, versus 40.23°C for the best model.

Figure 2(a) shows the value of the objective function for the best PLS model at each iteration for the WAAC algorithm compared to a single optimisation phase without any winnowing, Figure 2(b). The same random seeds are used for corresponding repetitions of the experiments, to ensure that the effect observed is not due to different initial models. In the absence of winnowing, premature convergence occurs and poorer solutions are found. This is also the case for the best SVM model shown in Figure 2(c) and 2(d).

**Figure 6:**
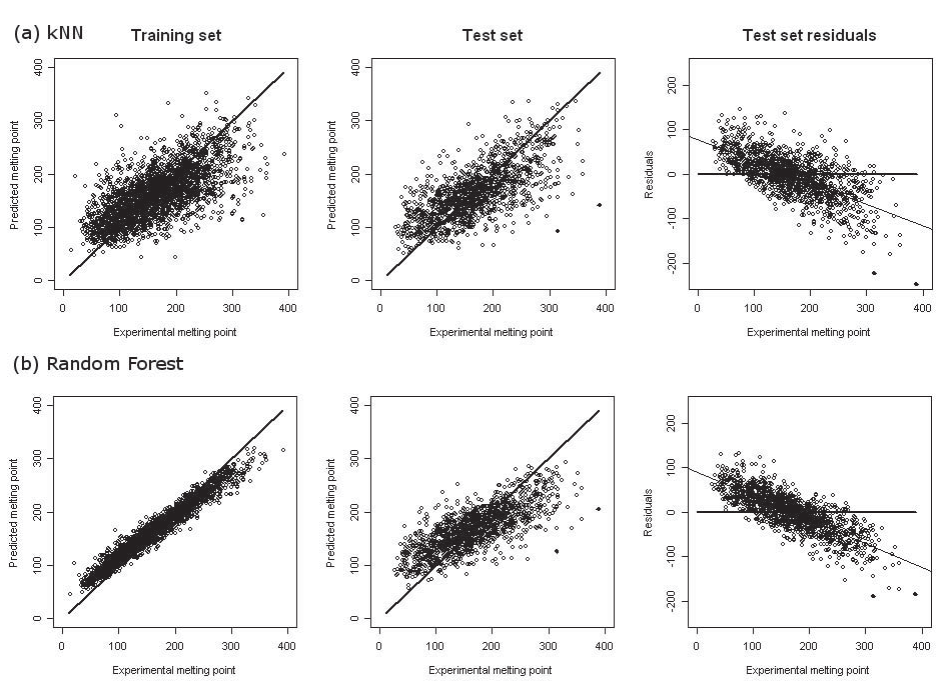
**Performance of (a) a *k*NN model, and (b) a Random Forest model**. The first two columns contain predictions for the training set and test set, respectively. The line *x *= *y *is shown for comparison. The column on the right shows the residuals from the test set prediction along with a line of best fit (light line); for comparison, the line *x *= 0 is shown (heavy line). Outliers are shown as filled circles in the test set prediction and residuals columns. All values in °C.

The Random Forest (RF) and *k*NN models for the same data are shown in Figure 6 and Table 1. Although performance on the training set does not give any indication of predictive ability, it is interesting to note how the different models have completely different RMSE(tr) and R^2^(tr). Performance on the external test set, which was not used to derive any of the models, allows us to assess predictive ability. On the basis of RMSE(ext), the RF model (44.5°C) is as good as, or slightly better than, the WAAC/SVM model (45.1°C), followed by the WAAC/PLS model (46.6°C) and then the *k*NN model (48.3°C). A similar order of predictive ability is shown by R^2^(ext), (RF: 0.55, WAAC/SVM: 0.54, WAAC/PLS: 0.51, *k*NN: 0.47). The bias shows a slightly different order for the two WAAC-derived models (RF: -0.4°C, WAAC/PLS: -0.7°C, WAAC/SVM: -2.1°C, *k*NN: -4.1°C).

However, looking at the test set predictions in the second column of Figures 3 and 6 it is clear, particularly for the RF model, that a systematic error occurs at the extremes of the melting point values in the dataset: low values are systematically overpredicted, while high values are underpredicted. In order to quantify the extent of this problem, we plotted the test set residuals versus the experimental melting point, and used linear regression to find the line of best fit (shown in the third column in Figures 3 and 6). For a model without this type of predictive bias, the expected slope is 0. The WAAC/SVM model performs best with a slope of -0.43, followed by the *k*NN and WAAC/PLS models which both have slopes of -0.49, while the RF model has a slope of -0.53. The standard errors of all of these values are 0.01.

Another effect of this systematic error is that the predicted values are bunched closer around the mean than the experimental values. The mean and standard deviation of the experimental values in the test set are 167.3°C and 66.4°C, respectively. All of the model predictions have a similar mean: 166.5, 165.2, 167.0 and 163.2°C for the WAAC/PLS, WAAC/SVM, RF and *k*NN models respectively. However, for the RF model the standard deviation of the predicted values is much smaller than that of the other models: 47.1, 51.6, 41.0 and 49.5°C for the WAAC/PLS, WAAC/SVM, RF and *k*NN models respectively.

**Figure 7:**
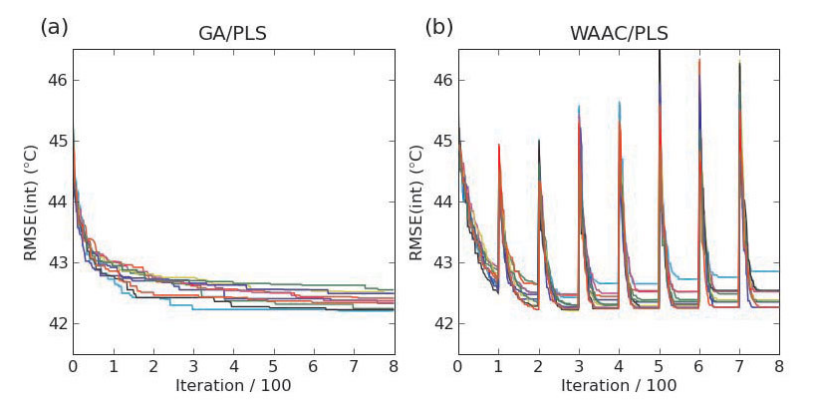
**Value of the objective function for the best PLS model at each iteration of (a) a genetic algorithm and (b) the WAAC algorithm**. Ten repetitions of each algorithm are shown. The number of PLS components was set to 49.

Another widely used stochastic method for feature selection is a genetic algorithm (GA). Hasegawa et al. [35] were one of the first to use a GA in combination with a PLS model to perform feature selection. The performance of the GA for feature selection is shown in Figure 7 compared to the WAAC algorithm. For both algorithms, the number of PLS components was fixed at 49. Convergence is much slower for the GA algorithm. In addition, the model with the fewest number of descriptors from 10 repetitions of each algorithm had 95 descriptors in the case of GA/PLS (objective function of 42.6°C) but only 57 for WAAC/PLS (objective function value of 42.3°C).

## Discussion

The development of the WAAC algorithm arose from an attempt to overcome the limitations of the modified ACO and ANTSELECT algorithms. Both of these algorithms determine probabilities by summing weights based on fitness scores. However, we observed that as convergence is achieved the fitness scores of the ant models in a particular iteration differ very little from each other. Thus, WAAC uses the fraction of the number of ants that have chosen a particular descriptor rather than a function of the fitness of the ants that have chosen that feature. Another problem with the use of weights is that they increase monotonically over the course of the algorithm whereas the sum of the number of ants has a clear bound. In addition, WAAC uses a value for ρ of 1, that is, complete evaporation. Values less than 1 were found to delay convergence without any corresponding improvement in the result. This makes sense when we consider that the evaporation parameter is supposed to help strike a balance between exploitation of information on previous models (global search) and exploration of local feature space (local search). However, this aspect is already included in Shen et al.'s algorithm and WAAC by the influence of the best models (global search) and current models (local search) on the moving probabilities. As a result of this simpler approach, the moving probabilities now have a meaningful interpretation: the probability of choosing a particular descriptor in the next iteration is equal to the average of the fraction of ants that have chosen that descriptor in their current model and the fraction of ants that have chosen it in their best model.

**Figure 8:**
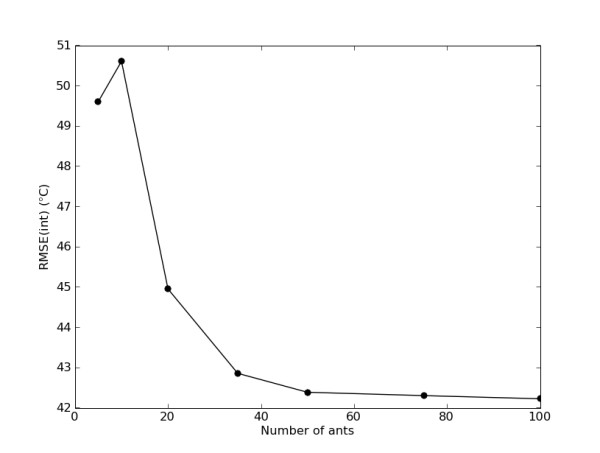
**Relationship between the population size and the minimum value of the objective function for the WAAC/PLS model**. The value of the objective function is the minimum found from ten repetitions of the algorithm.

Since the WAAC algorithm requires a range of allowed parameter values for the model, it is generally worthwhile to do an exploratory run of the algorithm to determine reasonable values. In addition, it is important that the number of allowed values for each parameter is less than the number of ants (preferably much less) to ensure that the parameter space is adequately sampled. An appropriate size for the ant population depends on the number of descriptors and the extent of the interaction between them. Model space will be better sampled if more ants are used, but the calculation time will also increase. However, since the feature-selection space is of size 2^n^-1, where *n *is the number of descriptors, the exact number of ants is not expected to affect the ability of the algorithm to find solutions. An ant population of between 50 and 100 ants is recommended. For the WAAC/PLS study, the relationship between the population size and the best value of the objective function is shown in Figure 8; there is little improvement beyond 50 ants. The length of the optimisation phase should be sufficient to allow the objective function to start to converge to an optimum value. It is not necessary to allow the optimisation phase to proceed much further, as after this point the descriptors chosen in the best models reinforce themselves and broad sampling of the search space no longer occurs. The winnowing procedure and subsequent reinitialisation on a smaller search space is a more effective way of finding the optimum model.

In the past, the development and comparison of feature selection methods for QSAR have involved the use of a standard dataset first reported in 1990, the Selwood dataset [36] of the activity of 31 antifilarial antimycin analogues, whose structures are represented by 53 calculated physicochemical descriptors. However, comparisons between different algorithms have been hampered by the fact that many of the descriptors are highly-correlated, and in addition, a true test using an external test set is not feasible due to the small number of samples. Advances in computing power mean that it is no longer appropriate to use such a small dataset for the purposes of testing feature selection algorithms. The Karthikeyan dataset used here is much more representative of the feature selection problems that occur in modern QSAR and QSPR studies.

PLS models are prone to overfitting. Figure 5 shows a comparison between a PLS model that uses the best subset (as selected by WAAC) and one using all of the descriptors. It is clear that the development of a predictive PLS model requires a variable selection step. Even if the number of components is optimised, performance is significantly poorer if all features are used instead of just the subset selected by the WAAC algorithm. It is also worth noting that PLS is a linear method, whereas SVM is a non-linear method. If the underlying link between descriptor values and the melting point cannot be adequately described by a linear combination of descriptor values, then the performance of the PLS method is likely to suffer. This may explain why, despite containing fewer than half the number of descriptors, the SVM model performed better than the PLS model.

Although the WAAC algorithm is capable of simultaneously optimising the feature selection as well as the parameter values, in some instances it may be preferable to use the WAAC algorithm simply for feature selection and optimise the parameter values separately for each model. This will only be computationally feasible where the model has a small number of parameters which need to be optimised and where the parameter optimisation can be efficiently carried out. For example, the optimal number of components for a PLS model could be determined by internal cross validation. When compared to the use of a genetic algorithm for optimising the feature selection of a PLS model, the WAAC algorithm performs well, both in terms of faster convergence and in its ability to produce models with fewer descriptors. It should be noted, however, that genetic algorithms have many different implementations as well as several parameters. This result, on a single dataset, cannot therefore be seen as conclusive.

In comparison to PLS models, the inclusion of a large number of descriptors does not necessarily lead to overfitting for SVM models. Although both Guyon et al. [14] and Fröhlich et al. [15], for example, have developed descriptor selection methods for SVM, an SVM model built on the entire set of descriptors and using the optimized parameters from the WAAC algorithm actually performs slightly better on the external test set. Here, the main effect of the WAAC algorithm is the identification of a minimum subset of descriptors which are the most important for the development of a predictive model. Such a procedure is especially useful when the descriptor values are derived from experimental measurement or require expensive calculation (for example, those derived from QM calculations). It also aids interpretability of the results.

Of the 28 descriptors selected by the WAAC/SVM model, three-quarters are 2D descriptors. Of these, many involve the area of the van der Waals surface associated with particular property values. For example the PEOE_VSA+2 descriptor is the van der Waals surface area (VSA) associated with PEOE (Partial Equalisation of Orbital Electronegativity) charges in the range 0.10 to 0.15. Also selected were descriptors relating to hydrophobic patches on the VSA (SlogP_VSA1, for example), the contribution to molar refractivity (SMR_VSA2, for example) which is related to polarisability, and the polar surface area (TPSA). Since the intermolecular interactions in a crystal lattice are dependent on complementarity between the properties of the VSA of adjacent molecules, the selection of these descriptors seems reasonable. Two descriptors were selected relating to the number of rotatable bonds (b_1rotR and b_rotR). These properties are related to the melting point through their effect on the change in entropy (ΔS_fus_) associated with the transformation to the solid state. Hydrogen bonds make an important energetic contribution to the formation of the crystal structure. This probably explains the selection of the descriptor for the number of oxygen atoms (a_nO), although strangely the number of nitrogen atoms is not included (it was however included in five out of the ten ant models). Four descriptors were selected by all ten ant models: b_1rotR, SlogP_VSA1, PEOE_VSA-6 and balabanJ. Balaban's *J *index is a topological index that increases in value as a molecule becomes more branched [37]. It seems possible that increased branching makes packing more difficult, and leads to lower melting points.

The WAAC algorithm appears to be robust to the presence of highly correlated descriptors. Despite the fact that such descriptors were not filtered from the dataset, the selected WAAV/SVM model contains only two pairs of descriptors with an absolute Pearson correlation coefficient greater than 0.8: b_rotR/b_1rotR (0.97) and SMR_VSA2/PEOE_VSA-5 (0.81). If the WAAC algorithm were unable to filter highly correlated descriptors, we would expect to see many more correlations as 16 of the chosen descriptors were highly correlated (absolute value greater than 0.8) with at least one descriptor not included in the final model. For example, radius has a correlation of 0.86 with respect to diameter (not unexpectedly). weinerPol is highly correlated with 35 other descriptors, none of which were chosen in the final model. PM3_LUMO is correlated with both AM1_LUMO (0.97) and MNDO_LUMO (0.96), but neither of other two appear.

For a small number of molecules, our models make very poor predictions. This may either be due to a lack of sufficient training molecules with particular characteristics, or it may be due to a fundamental deficiency in the information used to build the models. For example, for the WAAC/SVM models, three outliers can be detected whose residuals are more than four standard deviations from the mean (Figures 3 and 4). A polyfluorinated amide, *mol41*, is predicted to have a melting point of 233°C although its experimental melting point is 44°C. The melting points of the other two outliers were both underestimated: *mol4161*, m.p. 314.5°C but predicted 119°C, and *mol4195*, m.p. 342°C, but predicted 111°C. Both of these molecules have extended conjugated structures, causing the molecule to be planar over a wide area, and which are likely to give rise to extensive π-π stacking in the solid state. As a result, they are conformationally less flexible than might be expected from the number of rotatable bonds. *mol4161 *is also an outlier to the other three models; for WAAC/PLS it is the only outlier, whereas the RF and *k*NN predictions have a second outlier, *mol4208 *(Figure 4).

The WAAC algorithm described here is particularly useful when a machine learning method is prone to overfitting if presented with a large number of descriptors, such as is the case with PLS. However, not all machine learning methods require a prior feature selection procedure. The Random Forest (RF) method of Breiman uses consensus prediction of multiple decision trees built with subsets of the data and descriptors to avoid overfitting. For comparison with the WAAC results, we predicted the melting point values for the external test data using an RF model built on the training data. We also compared to a 15 Nearest Neighbour model (*k*NN) where the predictions of the set of neighbours were combined using an exponential weighting. In our comparison, the RMSE(ext) and R^2^(ext) show that the RF and WAAC/SVM models are very similar, and are better than the WAAC/PLS and *k*NN models. However, analysis of the residuals shows that the RF is more prone to bias at high and low values of the melting point compared to the other models.

A predictive bias was observed for all models at the extremes of the range of melting points. A similar effect was observed by Nigsch et al. for a kNN model of melting point prediction [33]. The effect was attributed to the fact that the density of points in the training set is less at the extremes of the range of melting point values. This means that the nearest neighbours to a point near the extreme are more likely to have melting points closer to the mean. This effect is most pronounced for the RF model, and the explanation may be similar.

In this study the WAAC algorithm was guided using the RMSE of prediction for an internal test set, RMSE(int). The choice of which objective function to use should be considered carefully. If an objective function is chosen which does not explicitly penalise the number of descriptors but only does so implicitly (for example, RMSE(int)), irrelevant descriptors may accumulate in the converged model. When using such an objective function, the winnowing procedure implemented in WAAC plays an important role in removing these descriptors after the optimisation phase by initiating a new search of a reduced feature space which makes it less likely that irrelevant descriptors will be selected. This effect is shown in Figure 2(b) and 2(d), where poorer models were found when the WAAC feature selection and parameter optimisation procedure was applied without winnowing.

An alternative type of objective function is one that explicitly penalises the number of descriptors. Such functions typically contain a cost term which is adjusted based on some *a priori *knowledge of the number of descriptors desired in the model. For example, the modified ACO algorithm of Shen *et al. *[26] was guided by a fitness function with two terms, one relating to the number of descriptors and the other to the fit of the model to the training set. Objective functions such as this quickly force models into a reduced feature space by favouring models with fewer descriptors. However, the moving probabilities used to choose descriptors will be misleading as they will largely be based on those descriptors present in models with fewer descriptors rather than those with the best predictive ability. As a result, descriptors with good predictive ability may be removed by chance. It should be noted that an objective function that simply optimises a measure of fit to the training data is not a suitable choice for the development of a model with predictive ability. Optimising the RMSE on the entire training data, RMSE(tr), or optimising the R^2^(tr) value, will produce an overfitted model that fits the training data exceptionally well but performs poorly on unseen data.

Near the end of each optimisation phase, the majority of ants converge to the same feature selection and parameter values, causing the same model to be repeatedly evaluated. It should be possible to gain a significant speedup if instead of re-evaluating a model, a cached value were used. Caching could be simply done by storing the objective function and models for all of the ants from the last few iterations. This is especially important if an objective function is used whose value varies on re-evaluation as is the case, for example, with the RMSE from n-fold cross-validation, RMSE(cv). Since for each ant the best score is retained, the value of the objective function will tend towards the optimistic tail of the distribution of values of the RMSE(cv). However, it should not have a major effect on the results of the feature selection and parameter optimisation, as model re-evaluation generally occurs only once the majority of the ants' models have already converged.

## Conclusion

The key elements to developing an effective QSPR model for prediction are accurate data, relevant descriptors and an appropriate model. Where there is no *a priori *information available on relevant descriptors, some form of feature selection needs to be performed.

We have presented WAAC, an extension of the modified ACO algorithm of Shen et al. [26], which can perform simultaneous optimisation of feature selection and model parameters. In addition, the moving probabilities used by the algorithm are easily interpreted in terms of the best and current models of the ants, and our winnowing procedure promotes the removal of irrelevant descriptors.

We have shown that the WAAC algorithm can be used to simultaneously optimise parameter values and the selected features for PLS and SVM models for melting point prediction. In particular, the resulting SVM model based on 28 descriptors performed as well as a Random Forest model that used the entire set of 203 descriptors.

## Authors' contributions

NMOB conceived and developed the WAAC algorithm, applied it to the melting point dataset, analysed the results and drafted the manuscript. DSP was involved in the interpretation of the results, revising the manuscript and carried out the Random Forest calculations. FN implemented the kNN model. JBOM contributed to the analysis of data and revising the manuscript. All authors read and approved the final manuscript.

## Supplementary Material

Additional file 1 **The external test set**. The models were evaluated by testing on this external test set. Click here for file

Additional file 2 **The internal training set**. The WAAC feature selection algorithm was trained on this. Click here for file

Additional file 3 **The internal test set**. The objective function used to guide the WAAC feature selection algorithm was calculated using this internal test set. Click here for file
